# Adaptation to Varying Salinity in *Halomonas elongata*: Much More Than Ectoine Accumulation

**DOI:** 10.3389/fmicb.2022.846677

**Published:** 2022-03-30

**Authors:** Karina Hobmeier, Martina Cantone, Quynh Anh Nguyen, Katharina Pflüger-Grau, Andreas Kremling, Hans Jörg Kunte, Friedhelm Pfeiffer, Alberto Marin-Sanguino

**Affiliations:** ^1^Systems Biotechnology, Technical University of Munich, Garching, Germany; ^2^Division Biodeterioration and Reference Organisms, Bundesanstalt für Materialforschung und -prüfung (BAM), Berlin, Germany; ^3^Computational Biology Group, Max Planck Institute of Biochemistry, Martinsried, Germany; ^4^Departament de Ciències Mèdiques Bàsiques, Universitat de Lleida, Lleida, Spain

**Keywords:** *Halomonas elongata*, ectoine, halophiles, transcriptomics, microbiology, biochemistry

## Abstract

The halophilic γ-proteobacterium *Halomonas elongata* DSM 2581^*T*^ thrives at salt concentrations well above 10 % NaCl (1.7 M NaCl). A well-known osmoregulatory mechanism is the accumulation of the compatible solute ectoine within the cell in response to osmotic stress. While ectoine accumulation is central to osmoregulation and promotes resistance to high salinity in halophilic bacteria, ectoine has this effect only to a much lesser extent in non-halophiles. We carried out transcriptome analysis of *H. elongata* grown on two different carbon sources (acetate or glucose), and low (0.17 M NaCl), medium (1 M), and high salinity (2 M) to identify additional mechanisms for adaptation to high saline environments. To avoid a methodological bias, the transcripts were evaluated by applying two methods, DESeq2 and Transcripts Per Million (TPM). The differentially transcribed genes in response to the available carbon sources and salt stress were then compared to the transcriptome profile of *Chromohalobacter salexigens*, a closely related moderate halophilic bacterium. Transcriptome profiling supports the notion that glucose is degraded *via* the cytoplasmic Entner-Doudoroff pathway, whereas the Embden-Meyerhoff-Parnas pathway is employed for gluconeogenesis. The machinery of oxidative phosphorylation in *H. elongata* and *C. salexigens* differs greatly from that of non-halophilic organisms, and electron flow can occur from quinone to oxygen along four alternative routes. Two of these pathways *via* cytochrome bo' and cytochrome bd quinol oxidases seem to be upregulated in salt stressed cells. Among the most highly regulated genes in *H. elongata* and *C. salexigens* are those encoding chemotaxis and motility proteins, with genes for chemotaxis and flagellar assembly severely downregulated at low salt concentrations. We also compared transcripts at low and high-salt stress (low growth rate) with transcripts at optimal salt concentration and found that the majority of regulated genes were down-regulated in stressed cells, including many genes involved in carbohydrate metabolism, while ribosome synthesis was up-regulated, which is in contrast to what is known from non-halophiles at slow growth. Finally, comparing the acidity of the cytoplasmic proteomes of non-halophiles, extreme halophiles and moderate halophiles suggests adaptation to an increased cytoplasmic ion concentration of *H. elongata*. Taken together, these results lead us to propose a model for salt tolerance in *H. elongata* where ion accumulation plays a greater role in salt tolerance than previously assumed.

## 1. Introduction

*Halomonas elongata* is a gammaproteobacterium, which was isolated from a solar saltern in the Netherlands Antilles (Vreeland et al., 1980). As a moderate halophilic organism, *H. elongata* can grow at salt concentrations well above 10 % NaCl. To cope with the considerable osmotic stress in brines and to maintain an osmotic equilibrium with the saline environment, *H. elongata* keeps its cytoplasm largely free of ions (Na^+^, Cl^−^) and instead accumulates small organic compounds in the cytoplasm (Wohlfarth et al., 1990). These compounds are uncharged but polar and highly soluble in water and do not interfere with cell metabolism, even at high cytoplasmic concentrations, which has earned them the name compatible solutes (Brown, 1976). Compatible solutes are quite diverse in nature and include sugars, polyols, and amino acids and their derivatives (Da Costa et al., 1998; Oren, 2008; Klähn and Hagemann, 2011; Jehlička et al., 2012). *H. elongata*, like many other halophiles, synthesizes the aspartate derivative ectoine (1,4,5,6,tetra-2-methyl-4-pyrimidinecarboxylic acid) as its main compatible solute (Galinski et al., 1985; Severin et al., 1992). Ectoine is synthesized starting from L-aspartate semialdehyde *via* diaminobutyric acid and N-acetyldiaminobutyric acid, and the corresponding synthesis enzymes are encoded by the gene cluster *ectABC* (Peters et al., 1990; Schwibbert et al., 2011). For energetic reasons, uptake of compatible solute from the medium is preferred over *de novo* synthesis (Oren, 1999). Uptake of ectoine from the medium is facilitated by the osmoregulated TRAP transporter TeaABC (Grammann et al., 2002), which has a high specificity for its substrates ectoine and hydroxyectoine (Kuhlmann et al., 2008). The TeaABC transport activity is modulated by ATP-binding protein TeaD, which belongs to the universal stress protein superfamily (Schweikhard et al., 2010). Ectoine uptake and its synthesis are interconnected, and it is postulated that a regulatory loop of ectoine release from the cell and subsequent uptake controls ectoine synthesis (Kunte, 2006). The importance of ectoine accumulation (by synthesis and transport) for osmoadaptation is demonstrated by the fact that ectoine-deficient mutants of *H. elongata* can no longer tolerate salt concentrations above 3 %. The importance is also evidenced by the fact that a non-halophilic bacterium such as *Escherichia coli* can increase its salt tolerance from about 3 % NaCl up to 6 % NaCl by ectoine accumulation. However, this increase in salt tolerance is miniscule compared to the broad salt tolerance of *H. elongata* and other halophiles. Clearly, halophilic bacteria must have additional osmoregulatory mechanisms other than compatible solute accumulation that give them that extra advantage. Long-known osmoregulatory mechanisms include changes in lipid composition with increasing salinity. Genome sequencing studies have shown that *H. elongata* and other halophilic bacteria possess numerous and distinct sodium efflux pumps compared to non-halophiles (Pfeiffer et al., 2017).

Although the metabolic capabilities of *H. elongata* are by now well known, there are still many open questions about how, where and when they are used. According to its genome, this bacterium has a large number of alternative ways to accomplish the same goal. It has a highly branched electron transport chain and its genome encodes genes to carry out four different types of glycolysis: the Embden-Meyerhoff-Parnas the pentose phosphate pathway and two alternative Entner-Doudoroff pathways, one periplasmic with non-phosphorylated intermediates and one cytoplasmic with phosphorylated compounds. Moreover, recent systems biology analyses have revealed the different course of anaplerotic pathways and the way they are energized through sodium gradients (Hobmeier et al., 2020). The aim of this research is to establish how *H. elongata* uses its rich metabolic toolset and to shed some light on how all these different parts are integrated to enable this bacterium to thrive at different salinities.

## 2. Materials and Methods

### 2.1. Strains and Growth Conditions for RNA-Seq Cultures

The strains used in this work are *H. elongata* DSM 2581^*T*^ wild type and strain KB2.13 (Δ*teaABC* Δ*doeA*) derived from the wild type, a strain unable to degrade ectoine, which also excretes it to the medium (Kunte et al., 2002). The cultures used for RNA sequencing were grown in three steps, always at 30°C and under shaking at 220 rpm. Initially, a single colony of the relevant strain was picked from an agar plate and used to inoculate 3 mL of LB medium with 1 M NaCl. This culture was grown over night and an aliquot was used as inoculum for the subsequent minimal medium pre-culture to an OD_600_ of 0.01. The 5 mL minimal medium MM63 (Larsen et al., 1987) was supplied with varying salt concentrations and 27.75 mM of different carbon sources depending on which growth condition was tested. All conditions are listed in Table 1 below. The pre-cultures were incubated until reaching an OD_600_ of approximately 1.0. An aliquot was then taken to inoculate the main culture in 500 mL shake flasks filled with 50 mL of the same medium used in the respective pre culture again to an OD_600_ of 0.01. The evolution of biomass in the main culture was monitored by measuring the OD_600_ in a spectrophotometer (Eppendorf, Germany). For each condition three biological were inoculated from different single colonies and grown in parallel. Also, notably, the cultures were grown in two separate batches with the low salt and high salt condition in a first experiment and the remaining conditions in a second set.

**Table 1:** Summary of all different combinations of strains, carbon sources and salt concentrations used for the RNA sequencing.

**Condition**	**Strain**	**Salt [M]**	**Carbon source**
Low salt	Wild type	0.17	glucose
High salt	Wild type	2.0	glucose
Salt optimum	Wild type	1.0	glucose
Gluconeogenesis	Wild type	1.0	acetate
strain KB2.13	Δ*teaABC* Δ*doeA*	1.0	glucose

*Cultures of the Wild type and of the strain KB2.13 (ΔteaABC ΔdoeA) were grown in MM63 minimal medium with either different salt concentrations or a different carbon source (27.75 mM)*.

### 2.2. PHB Measurement

The formation of PHB granules was analyzed *via* nile red staining of the PHB granules (Gorenflo et al., 1999) and *via* HPLC analysis by conversion of PHB to crotonic acid (Karr et al., 1983).

The nile red stain, due to its lipophilic nature, is able to bind to the PHB polyesters which are accumulated as insoluble inclusion bodies in the cytoplasm. Using the emitted fluorescent signal of the nile red stain the presence and amount of PHB can be assessed. First, the living *H. elongata* cells were treated with a 1 mg/mL nile red stock solution (acetone) to an end concentration of 3 μg/mL. Then, the cells were examined under an Olympus IX70 microscope (Olympus, Japan) using a 60X objective and the nile red fluorescence was excited with a polychromator at a wavelength of 560 nm.

For the HPLC detection of PHB the biomass of 0.9 mL of the culture was digested in 0.25 mL concentrated sulfuric acid. After incubation for 1 h at 90°C the PHB is depolymerized, forming crotonic acid, which can be detected by HPLC at 210 nm. For the separation a reverse phase column was used (Kinetex 5 μm EVO C18 150/3, Phenomenex, USA) applying a 7 mM H_2_SO_4_ solution as mobile phase. The digested PHB samples were diluted 1:10 with mobile phase.

### 2.3. RNA Isolation and Sequencing

The cultures used for RNA sequencing were grown to reach the exponential phase and collected at an OD_600_ of approximately 0.5. For the first experiment including cultures grown at 0.17 M NaCl (low salt) and 2 M NaCl (high salt), 10 mL samples were taken and mixed 2:1 with RNAprotect reagent (Qiagen, Germany) which is used to stabilize the RNA and preserve the expression profile. Cells were harvested by centrifugation for 5 min at 5,000 rpm and at room temperature. In the second experiment including the remaining conditions RNAprotect reagent (Qiagen, Germany) was applied in a ratio 1:1 using 6 mL for cultures grown on acetate and 4 mL for those grown on glucose. The biomass in these samples was separated by centrifugation for 7 min at 5,000 rpm and at room temperature. The total RNA was extracted from the biomass samples following manufacturer's instructions for the Macherey-Nagel™NucleoSpin™RNA kit (Macherey Nagel, Germany) and sent to GATC Biotech company (Germany). Strand specific cDNA library generation and RNA sequencing were performed at the company facilities on Illumina HiSeq 4000 sequencer and Illumina NovaSeq 6,000 S4 XP for the first and second experiment respectively. The obtained reads for the first experiment were 50 bp long (single end) and for the second experiment 150 bp long (paired end).

### 2.4. Processing Sequencing Data

Quality control of the fastQ files was performed using FastQC (Bioinformatics, 2011). No trimming or filtering was necessary. The reads were aligned to the genome using Bowtie2 (Langmead and Salzberg, 2012) and the number of reads per gene was determine using SAMtools (Li et al., 2009). Raw counts were normalized for GC content using EDASeq (Risso et al., 2011). The counts were then analyzed using DESeq2 (Love et al., 2014b) and also converted to Transcript Per Million (TPM) using a Python script (Van Rossum and Drake, 1995).

### 2.5. Genomic Analysis

To determine the frequency of acidic and basic amino acids in the proteome of diverse bacteria, the translation of their respective CDS were downloaded from NCBI's Assembly database (Kitts et al., 2016) and a most probable location for each entry was determined using psortDB (Peabody et al., 2016). The isoelectric point calculations were done using biopython (Cock et al., 2009).

## 3. Results

### 3.1. Quantifying Transcription Levels

The gene expression profile of *Halomonas elongata* under different conditions was analyzed using RNA-Seq. The wild type was grown on glucose at low salt (0.17 M NaCl) and high salt (2 M NaCl). These concentrations were chosen for causing a very similar reduction on growth rate compared to optimal conditions, with cultures growing at 0.304±0.011*h*^−1^ at high salt and 0.353±0.005*h*^−1^ at low salt. This choice minimizes the impact of growth rate as a confounding variable and ensures that both cultures are under comparable levels of stress due to excess or lack of salt. In order to provide further insight on these results, another sequencing experiment was performed with wild type cultures growing at salt concentrations of 1 M NaCl (within the optimal interval) on glucose with growth rate (0.443±0.039*h*^−1^) and acetate with growth rate (0.244±0.037*h*^−1^). Moreover, cultures of the strain KB 2.13 (Δ*teaABCΔdoeA*) that excretes ectoine to the medium were also analyzed.

Two different approaches were used to analyze gene expression at the transcriptional level: the software DESeq2 (Love et al., 2014a) and counts normalized as Transcripts Per Million (TPM) (Li and Dewey, 2011; Wagner et al., 2012). Each approach has its strong points and scopes of application. DESeq2 is one of the standard tools for analyzing differential expression and uses shrinkage (Robinson and Smyth, 2007) to consider the variance of the whole experiment to assess the statistical significance of the expression changes for each gene. This enables the calculation of sharper p-values than would be possible considering the expression of each gene separately with only as many repeats as transcriptomics experiments. This approach also has some drawbacks. Data from different experiments cannot be compared, ranking of genes within a sample is not possible and the normalization method used might not be the most appropriate when studying prokaryotes. The latter can be better understood adapting a hypothetical scenario from (Robinson and Oshlack, 2010). Let us assume two samples in which gene A is transcribed to the same extent. But one of the samples is also expressing gene B, which is silent in the other. The count in TPM for gene A will be lower in the sample that also expresses gene B. This adjustment is what DESeq and most DE normalization methods try to avoid, since it would result in a large number of falsely differentially expressed genes when comparing different eukaryote tissues. In prokaryotes, however, key properties of a culture, such as growth rate, are precisely explained by changes in the fraction of the proteome that is occupied by certain gene products (Scott et al., 2010). Just as important as which genes that are taking up more space in the proteome because they are upregulated, is the question of which genes give away space by failing to keep up. An additional limitation of this type of normalization is the assumption of a symmetric degree of regulation when comparing two samples, with approximately the same number of genes being up- and down-regulated. This symmetry assumption might distort the results, when comparing high vs. low salt stress. An asymmetric response may indicate that the cell is better adapted to one scenario than to the other, and this would be masked by normalization. This is not to say that TPM is free of pitfalls. Comparing TPM between different experiments can be subject to a number of biases due to compartmentalization, library preparation protocols and different levels of total RNA (Zhao et al., 2020). Even though most of these cases only apply to eukaryotic samples, TPM count still has the drawback of not being compatible with shrinkage, so the only statistical test to determine significance is a gene by gene log-ratio t-test, which is not very meaningful for less than 12 replicates (Schurch et al., 2016). Due to the impossibility of finding a one-size-fits-all solution, we will apply both methods as being complementary to each other. From now on, a gene will be said to be differentially expressed between two conditions if this is backed by both methods unless otherwise specified. Comparisons including the transcription profile from different experiments—e.g., at low, optimal and high salt—will be based on TPM count, since DESeq is not meant to be used in such comparisons. The complete results of applying each method can be found in (Supplementary Table S1 for TPM, Supplementary Table S2 for high- vs. low salt, Supplementary Table S3 for WT vs. strain KB2.13 and Supplementary Table S4 for acetate vs. glucose).

**Figure 1:**
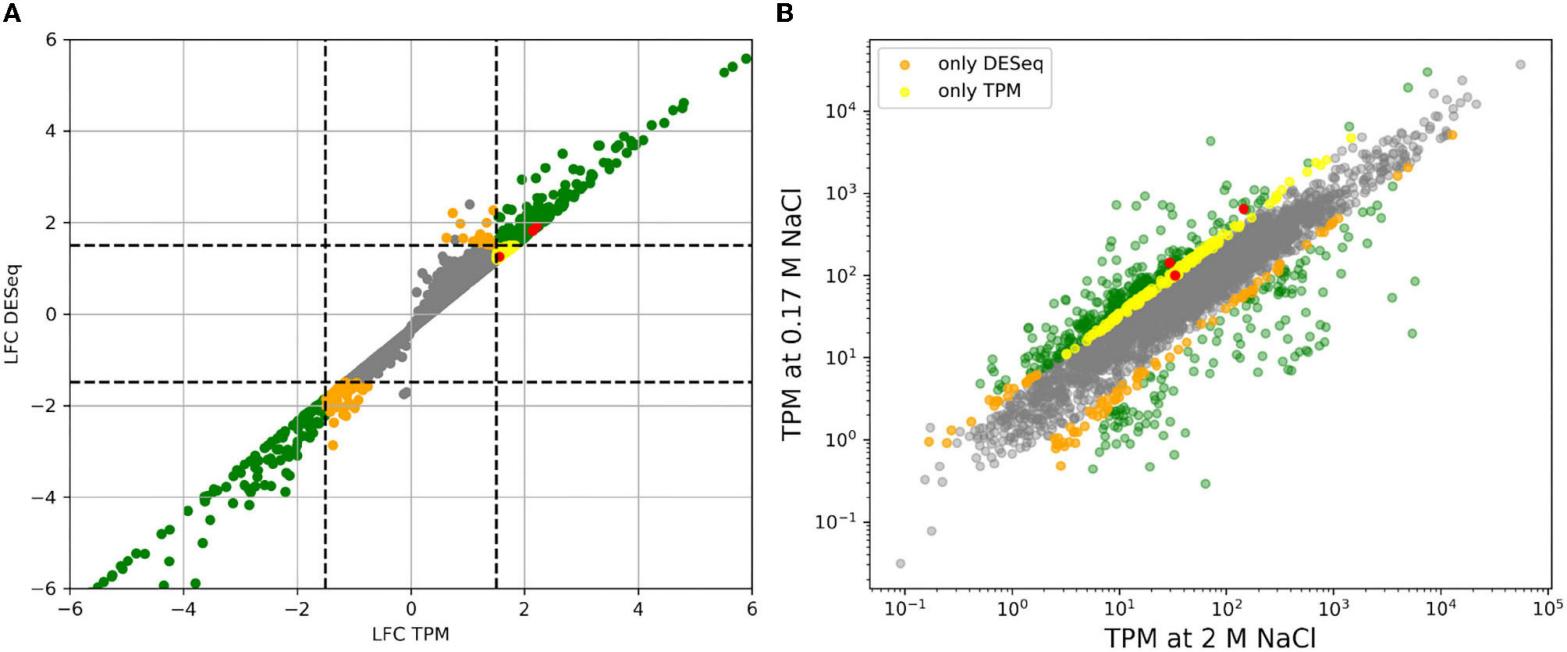
**(A)** Comparison of log2-fold-changes (LFC) between low salt (0.17 M NaCl) and high salt (2 M NaCl) obtained using TPM count vs. DESeq. Dashed lines mark the threshold for differential expression. **(B)** Comparison of TPM counts between high and low salt. Genes that are considered to be differentially expressed by both methods are plotted in green. Only by DESeq in orange and only by TPM count in yellow. The three genes for PHB synthesis have been taking as a reference and marked in red since accumulation of PHB granules is clearly higher in cells growing at low salt compared to those growing at high salt. See below.

**Figure 2:**
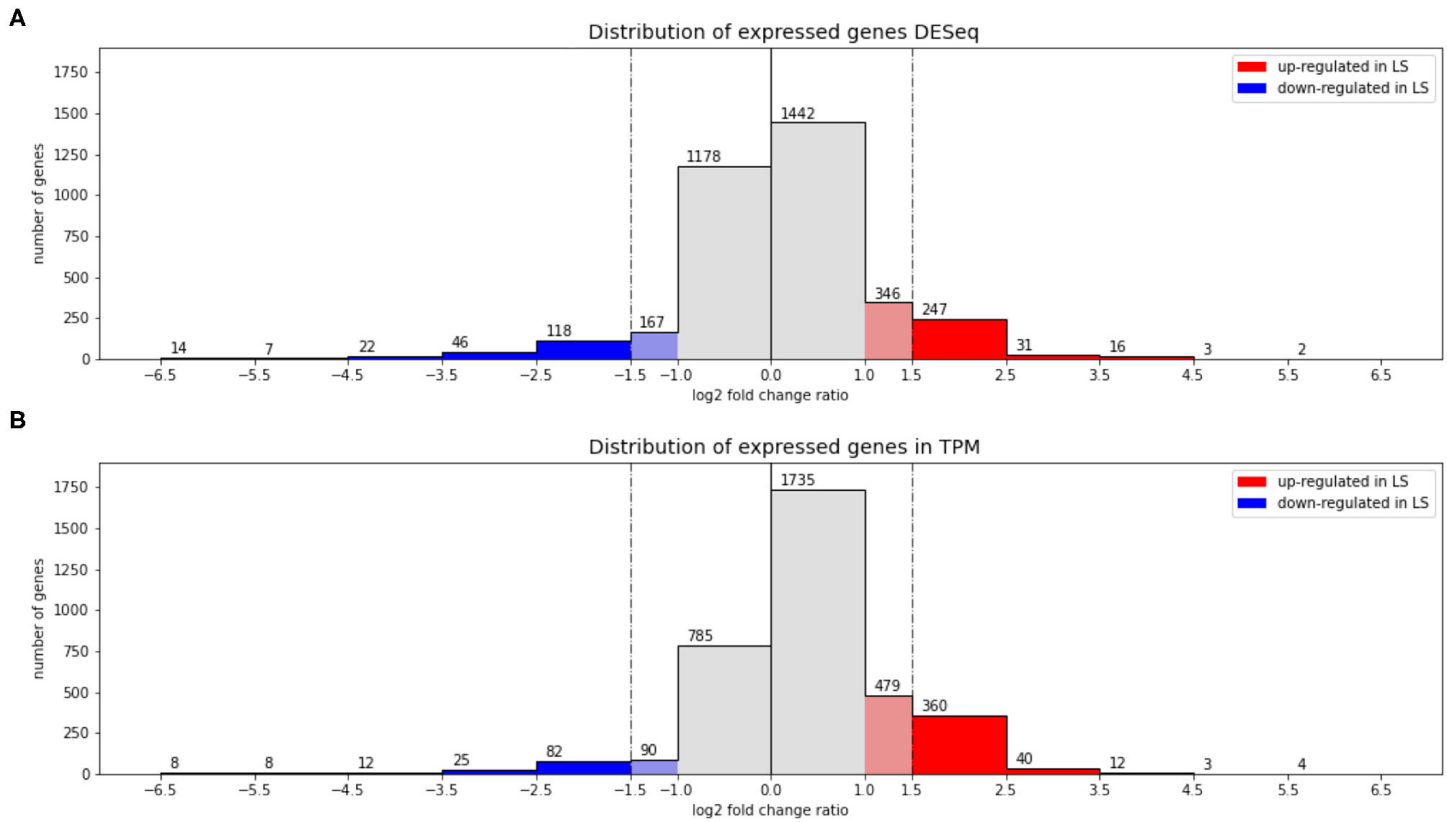
Asymmetry in low vs. high salt regulation. **(A)** Shows a histogram of log2-fold-changes obtained using DESeq and **(B)** the equivalent for TPM. Vertical lines show the chosen threshold to consider a gene differentially expressed (see text for details).

Figure 1 shows the result of applying both methods to the comparison of high vs. low salt growth. The log2-fold-changes of both DESeq and TPM count show moderately asymmetric data with skewness of −0.98 and −0.95, respectively. The differences become more pronounced after applying a threshold to define differential expression. DESeq2 predicts a total of 508 genes having an absolute log2-fold-change greater than 1.5 between low and high salt (299 up, 209 down, where up means preferentially expressed at low salt). Of these 508, 504 were determined to be statistically significant (*p*-value ≤ 0.05). Using TPM, 555 genes had absolute log2-fold-changes greater than 1.5 (419 up, 136 down). In both cases, we can see a certain asymmetry in the responses, even if it is inherently dampened in DESeq2. This can be clearly seen in Figures 1, 2.

The intersection of both methods indicates a total of 414 genes differentially expressed (278 up 136 down). 141 genes appear differentially expressed only according to TPM (all of them up) and 90 according only to DESeq2 (19 up, 71 down). So requesting double confirmation by both methods would result in a core of 414 clearly differentially expressed genes, while 231 are potentially regulated as pointed out by only one method.

**Figure 3:**
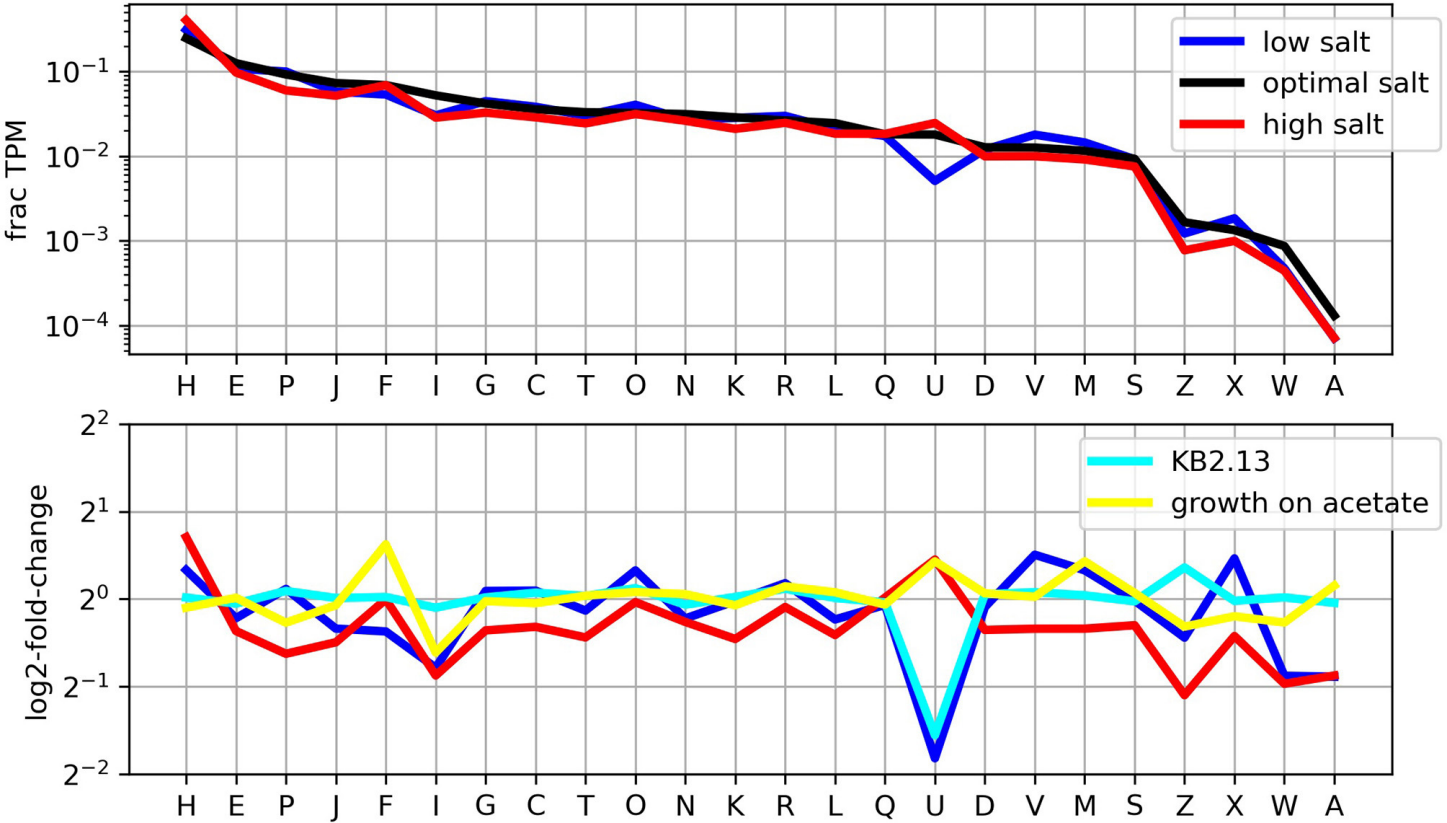
Distribution of transcription levels by COG classes. The **upper panel** shows the fraction of the transcriptome in TPM that corresponds to each COG class. The **lower panel** shows the (log) ratios of the frequencies for each condition compared to growth on glucose at optimal salt concentration.

Classification of transcription levels by COG class as shown in Figure 3 provides an overview of the importance of each cellular function in the different conditions studied. The most remarkable feature is the clear drop in the transcription of motility related genes (COG class N) at low salt and how this effect of low salt in the medium is reproduced at optimal salt in the strain KB2.13.

### 3.2. PHB Accumulation

**Figure 4:**
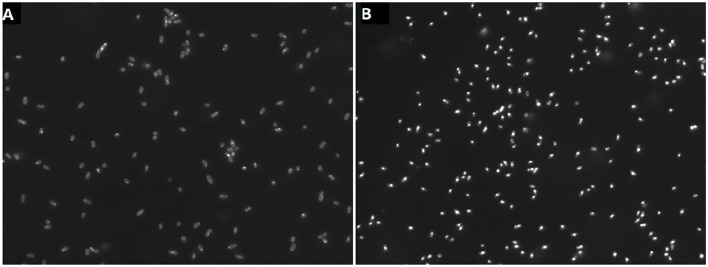
Cells grown at 2 M NaCl **(A)** and at 0.17 M NaCl **(B)** stained with nile red to show PHB granules.

**Figure 5:**
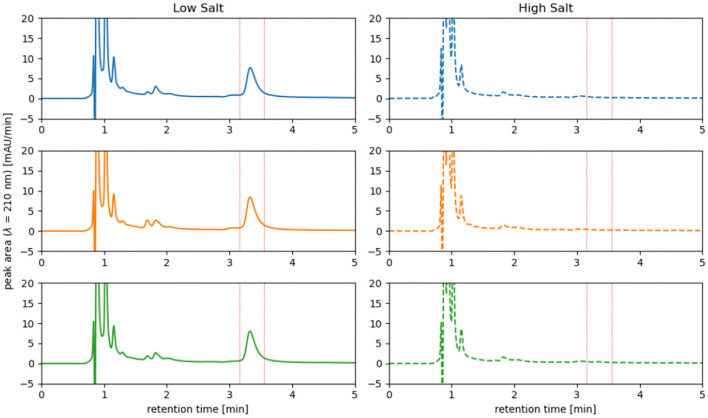
HPLC analysis of PHB accumulated in *H. elongata* wildtype in low salt condition (0.17 M NaCl) vs. high salt condition (2 M NaCl). At a retention time of approximately 3.25 min a distinct peak is visible for all three replicates grown at low salt while at high salt no peak can be observed. Analysis was performed by conversion of PHB into crotonic acid and detection at 210 nm.

Cells of *H. elongata* grown at high salt (2 M NaCl) concentration do not accumulate significant amounts of PHB, as can be seen in Figures 4, 5. However, cells grown at low (0.17 M NaCl) salt show a clear accumulation of PHB granules.

### 3.3. Correlation With Growth Rate

The expression of many genes is known to correlate with growth rate. This is, at least, the case for fast growing cultures (Scott et al., 2010). It is also known that proteome adjustments at slower growth rates are qualitatively different from those of fast growing cultures. Given the TPM counts for the wild type growing in the different conditions analyzed in this study: growth on glucose at 0.17, 1, and 2 M NaCl, as well as growth on acetate at 1 M NaCl, regression of gene TPM counts vs. growth rate showed that only 22 genes have a determination coefficient (R^2^) higher than 0.6. This is consistent with cells growing slowly on a defined medium, specially for the ribosomal genes, whose expression correlates with growth rates on fast growing cells but here show determination coefficients below 0.4 with a mean of 0.05 and standard deviation 0.07 (see Supplementary Table S5).

### 3.4. Effects of the Carbon Source

The comparison between transcription levels for the wild type growing on two different carbon sources, acetate and glucose, resulted in a total of 45 genes up-regulated on acetate and the same number on glucose (see **Supplementary Information** for details).

Unsurprisingly, a battery of sugar transporters was preferentially transcribed on glucose in comparison with growth on acetate. These genes include glucose, maltose and trehalose transporters and porins. Also upregulated on glucose are the genes HELO_1043 and HELO_1044 encoding for a tricarboxylic acid transport protein, while HELO_1625 that encodes the substrate-binding protein of a transporter of the same family is up-regulated on acetate. So is HELO_3259, which encodes a sodium-proline symporter and HELO_3085 which codes for a similar symporter of unknown substrate specificity.

Three genes involved in nitrogen metabolism are up-regulated in cells grown on acetate, namely HELO_2857 encoding for a transporter and two genes of the nitrate reductase cluster: *narGH* (HELO_2853-4). The same is observed with two genes in the cytochrome bd ubiquinol oxidase cluster *cydAB* (HELO_2456-7). Among the genes involved in amino acid metabolism, those upregulated on acetate include: *putA* (HELO_1802) coding for bifunctional proline dehydrogenase, *mmsA1* (HELO_1515), *mmsB* (HELO_2425), and HELO_2317 probably involved in the metabolism of aliphatic amino acids. On the other hand, genes *trpA1* and *trpB1* coding for a tryptophan synthase (HELO_4326-7) are upregulated on glucose.

The rest of the differentially expressed genes belong to the central pathways of carbon metabolism. The genes involved in the cytoplasmic (phosphorylated) version of the Entner-Doudoroff pathway are preferentially expressed on glucose: *zwf* (HELO_3637), *pgl* (HELO_3636), *edd* (HELO_3628), and *eda* (HELO_3635). Genes encoding glucose-6-phosphate isomerases *pgi1* (HELO_4245) and *pgi2* (HELO_1718), pyruvate kinase *pykA1* (HELO_4243) and alcohol dehydrogenase *adh2* (HELO_2818) are upregulated on glucose as well. Cells growing on acetate show an up-regulation of the glyoxylate shunt genes *aceA* (HELO_3070) and *glcB* (HELO_4288) as well as glyoxylate related glyoxylate carboligase *gcl* (HELO_1601), hydroxypyruvate isomerase *hyi* (HELO_1602) and *glxR1* (HELO_1603). Moreover, gluconeogenetic genes that are also up-regulated on acetate include the phosphoenolpyruvate carboxykinase *pckA* (HELO_1685) and phosphoenolpyruvate synthase *ppsA* (HELO_2433). Additionally, a homolog to glyceraldehyde 3-phosphate dehydrogenase (HELO_2214) was up-regulated on acetate as well.

### 3.5. Regulation by Salt and Comparison With *Chromohalobacter salexigens*

*C. salexigens* is a close relative to *H. elongata* (Arahal et al., 2001) and a recent transcriptomic study compared its gene expression profile between cells grown at high vs. low salt stress (Salvador et al., 2018). This provides the opportunity to analyze the similarities and differences between the salt response of two phylogenetically close organisms that also occupy very similar ecological niches.

A homology matrix computed between the two organisms according to the workflow presented by Norsigian et al. (2020) using a threshold of 30 % found 2,332 homologous loci. These were cross indexed with the loci classified as differentially expressed in Supplementary Tables S6–S11 of Salvador et al. (2018). Of 227 differentially expressed loci described in the tables, 177 had homologous genes in *H. elongata* (see Supplementary Table S6). For instance, out of the 30 loci related to ectoine and compatible solutes metabolism that were differentially expressed in *C. salexigens*, 24 had homologues in *H. elongata* and six of them (25 %) were differentially expressed in this study as well (see Table 2). Also worth notice are chemotaxis and motility genes, where more than 90 % of the homologous genes are differentially expressed in both organisms and protein folding stress genes, where even though 78 % of the genes differentially expressed in *C. salexigens* had homologues in *H. elongata*, only one of them was differentially expressed.

**Table 2:** Homologues in *H. elongata* of the genes found differentially expressed in *C. salexigens*, Supplementary Tables S6 to S11 in Salvador et al. (2018).

	**DE *C. salexigens***	**N homologues**	**DE both**	**DE only TPM**	**DE only DESeq**
Compatible solutes	30	24	6	1	3
Oxidative stress	42	29	3	3	0
Iron homeostasis	37	27	4	6	0
Oxidative phosphorylation and electrochemical gradients	55	41	3	4	1
Protein folding stress	19	15	1	2	0
Chemotaxis and motility	45	41	38	0	3

*For each group of genes found differentially expressed in C. salexigens, the first column lists how many genes of each group were reported as differentially expressed in C. salexigens, subsequent columns show: how many of those genes had homologues in H. elongata, how many of those were found to be differentially expressed according to both methods applied, which were only so according to DESeq and which only according to the TPM count. While DESeq was only applied to the difference between high and low salt, TPM counts were compared between all three conditions and a log2-fold-change above the threshold between any two, led to inclusion in the list*.

#### 3.5.1. Compatible Solutes

From a total of twenty-four genes in this group that had homologues differentially expressed in *C. salexigens*, six were classified as such in *H. elongata*. All three genes coding for the TRAP transporter for ectoine uptake *teaABC* are downregulated at low salt while keeping comparable levels of transcription at optimal and high salt. *H. elongata* has a fourth gene clustered with *teaABC, teaD*, which is absent in *C. salexigens* and appears upregulated at high salt according only to DESeq. Two genes of the ectoine degradation pathway (*doeAB*) show the same trend as in *C. salexigens*. The two first genes in the ectoine synthesis pathway (*ectAB*) are downregulated at low salt while the third (*ectC*) keeps being transcribed at a level that correlates with salt. The gene *ectD*, that codes for the hydroxylation of ectoine to hydroxyectoine, becomes upregulated only at high salt.

Other genes with related functions that were found to be differentially expressed are HELO_1580 (*betG*) and HELO_3358 (*betH*), two glycine betaine transporters of the BCCT family. While the first gene appears to decrease its transcription with increasing medium salinity, the second follows an opposite trend. A third gene, also part of a BCC transporter of undetermined substrate specificity (HELO_3694) was found to be downregulated at low salt.

#### 3.5.2. Oxidative Phosphorylation and Electro-Chemical Gradients

**Figure 6:**
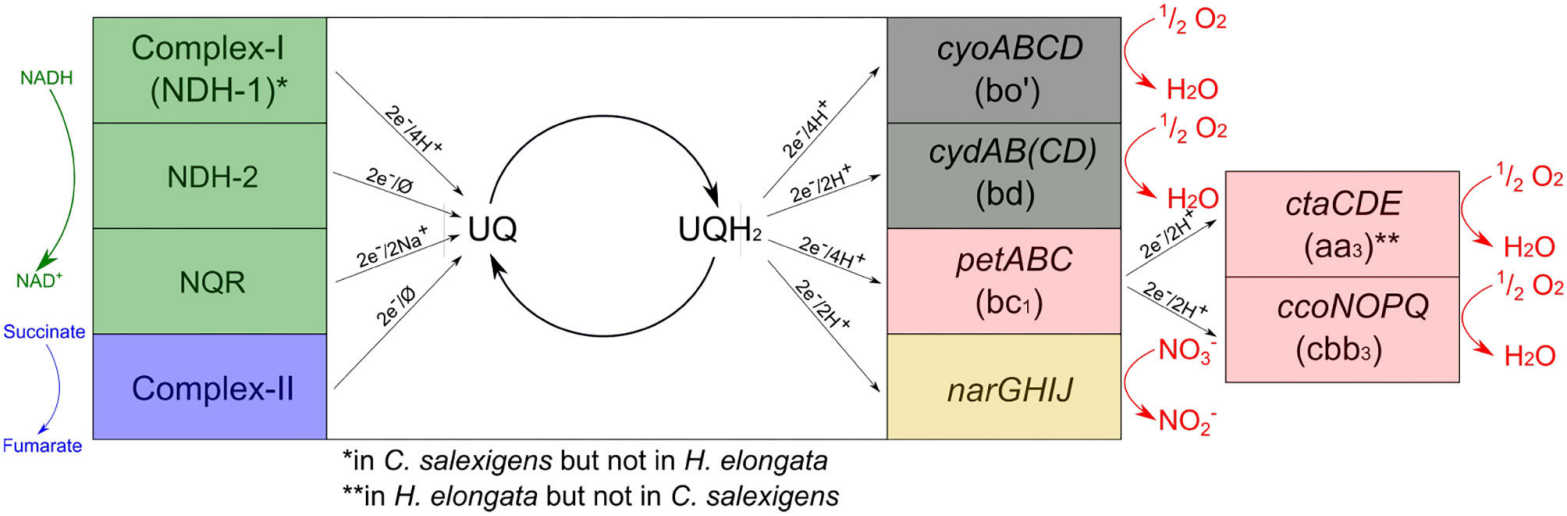
Overview of the electron transport chain of *H. elongata* including the ratio of protons (or sodium ions) transported per electron (∅ if no protons or sodium ions are pumped).

The machinery for oxidative phosphorylation in *H. elongata* (see Figure 6) has similarities to that of its close relative *C. salexigens* but also important differences. *H. elongata* does have a set of genes encoding for caa_3_ terminal oxidases (HELO_2502-10) but it does not have an NDH-1 analogous to the complex-I in mitochondria. Moreover, *H. elongata* has a V-type ATPase that is not present in *C. salexigens*. At the NADH dehydrogenase level, *H. elongata* has only a sodium-translocating Complex NQR (*nqrABCDEF*, HELO_2215-20) that translocates two sodium cations instead of protons and an NDH-2 (HELO_3377), which has no translocating activity at all. After electrons have reached the quinone level, the electron transport chain of *H. elongata* offers four alternative branches conducing toward oxygen. Two of these branches channel the electrons through the cytochrome bc1 complex (*petABC*, HELO_1943-45), which is analogous to mitochondrial Complex III, and then through one of two cytochrome c oxidases analogous to Complex IV: either the above mentioned caa_3_ oxidase, *ctaCDE* (HELO_2502-10) or the cbb_3_, *ccoNOPQ* (HELO_3536-39). These two pathways are similar to those found in the mitochrondria and are not available to *E. coli*, which can only channel the electrons further through cytochrome b quinol oxidases. Of these, *H. elongata* has two alternatives: One is a bo' quinol oxidase (*cyoABCD*, HELO_3152-55) similar to that used by *E. coli* under aerobic growth. This membrane protein is a proton pump with an H^+^/e^−^ ratio of 2. The second is a cytochrome bd quinol oxidase encoded by *cydAB(CD)*. It generates the proton motive force by releasing protons from quinol oxidation into the periplasm while taking the protons to form water from molecular oxygen from the cytoplasm (Skulachev, 1984). This results in a lower H^+^/e^−^ ratio of 1. In *E. coli* respiration *via* cytochrome bd is generally linked to growth in microaerobic conditions due to a higher affinity for oxygen compared to quinol oxidase bo' and is regulated by the transcriptional factor FNR among others (D'Mello et al., 1995; Shepherd and Poole, 2012). However, activity of quinol oxidase bd has not only been linked to low oxygen availability, but also to other stress conditions like high temperature, high pH or also membrane de-energization (Giuffrè et al., 2014). One distinct difference between the cytochrome oxidase types c and b is the type of bonds in the active sites. Since the c type cytochromes are located in the periplasm or loosely adsorbed on the outer side of the membrane the bonds in the active site are covalent due to the risk of losing the heme of the active site (Allen et al., 2003; Shepherd and Poole, 2012).

Additional dehydrogenases found in *H. elongata* include for instance the Complex II ortholog succinate dehydrogenase (*sdhABCD* HELO_3113-16), a formate dehydrogenase (HELO_1895-1899, formerly annotated as nuo) or lactate dehydrogenase (*lldD*, HELO_1220).

**Figure 7:**
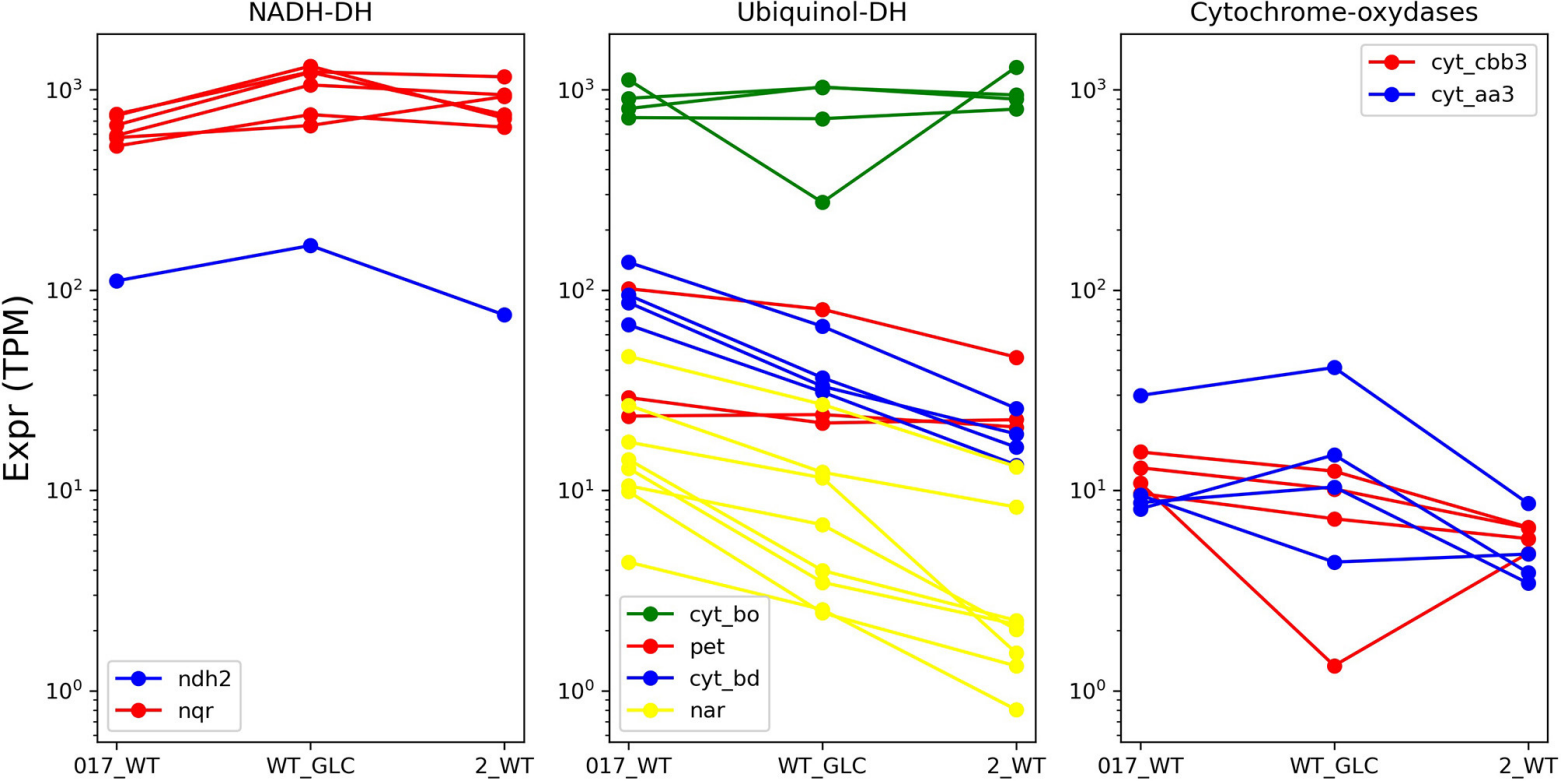
Transcription levels of the different branches of the electron transport chain at different levels.

The significant differences in the machinery of oxidative phosphorylation makes it unsurprising that the behavior of the corresponding genes differs between *H. elongata* and *C. salexigens* in spite of their phylogenetic proximity (see Figure 7). Even though 41 of the genes that were differentially expressed in *C. salexigens* have homologues in *H. elongata*, only three of these were found to be differentially expressed: a subunit of the F-type ATP synthase (HELO_4445) is upregulated at high salt and two genes of the bd quinol oxidase (HELO_2456 and HELO_2457) show negative correlation between transcription levels and salt. Three more genes are backed by only one method. By TPM count, a gene related to the same ATP synthase (HELO_4449) and the Mrp-type Na^+^/H^+^ exchanger (HELO_3518). According to DESeq, a gene probably related to K^+^ uptake (HELO_3371), is upregulated at high salt. Additionally, *trkI* (HELO_1450) shows the opposite trend according to TPM count.

The overall high level of transcription of the *cyoABCD* genes, clearly shows that, in agreement with *E. coli* growing aerobically, the quinol oxidase bo' (cytochrome c class IV) is the main pathway for electron flow among the four alternatives present in *H. elongata*. HELO_3152 in this complex is also overexpressed on both forms of salt stress. A similar albeit less clear trend is shown by HELO_3538 of the ccb_3_ oxidase complex, with the log2-fold-change between optimal and high salt falling closely short of the chosen threshold. The transcription of bd quinol oxidase (*cydABCD*) shows a negative correlation with salinity. This is counter-intuitive, since this pathway is associated with decreasing oxygen (microaerobic conditions) in *E. coli*. It is noteworthy that this trend is shared by HELO_2679, an UPF0057 family protein. Proteins of this family are present in all domains of life and their expression tends to increase with falling membrane potentials (Kwok et al., 2020), another known cause for overexpression of bd quinol oxidases. Additionally, two genes of the cytochrome caa_3_ oxidase (*ctaCD*) show similar transcription levels at low and optimal salt with a marked downregulation at higher salinity. It is noteworthy that all regulated genes seem to be concentrated at the lower electron transport chain. Genes for the quinol reductases (NDH-2 or NQR) that are known to be regulated by salt in related organisms (Chen et al., 2017; Salvador et al., 2018) keep constant levels of transcription under all conditions analyzed. It is also remarkable that all the genes for nitrate reductase show a consistent trend toward higher transcription levels at low salt, in spite of the absence of any nitrogen source other than ammonia in the medium. All subunits of the V-ATPase, are predicted to be differentially expressed by both TPM count and DESeq. Three of these genes are upregulated at low salt with respect to other salt concentrations and the rest show a steadily decreasing trend as salt increases.

Regarding the generation and maintenance of electrochemical gradients, six of the seven genes composing the Mrp1 system for Na^+^/H^+^ exchange are regulated by salt with three of them showing a general downward trend as salt increases while the other three show a sharp downregulation as salt increases from low to optimal. One of the genes of the similar Mrp2 K^+^/H^+^ exchanger (HELO_3518) is indicated by TPM count as upregulated under both salt stress conditions. Finally, another Na^+^/H^+^ exchanger, *nhaD1* (HELO_1427), shows differential expression between low and optimal salt in TPM count and is also pointed out as differentially expressed by DESeq. NhaD1 would be expected to have a different stoichiometry from Mrp1 and there are indications that it may be operating in the opposite direction (Kurz et al., 2006).

#### 3.5.3. Oxidative Stress

Of 29 genes homologous to regulated oxidative stress genes in *C. salexigens*, only three were differentially expressed according to both methods: DprA (HELO_1377) is a recombination mediator protein usually involved in transformation (Ando et al., 1999; Smeets et al., 2006), DsbA (HELO_4264) is a thiol-disulfide interchange protein and MsrB2 a methionine sulfoxide reductase (see below). The genes coding for all three proteins show steadily decreasing transcription as salt increases.

The transcription of enzymes involved in repair processes due to oxidative stress is similar to that in *C. salexigens* involving higher levels at low salt of two glutaredoxin-like enzymes as well as a thioredoxin-like and the *msrAB2* genes. The response regarding reactive oxygen species (ROS) scavenging enzymes is, however, distinct between the two bacteria. Neither the catalase nor the two superoxide dismutases were regulated by salt and the two peroxiredoxin candidates that were regulated (HELO_1409 and HELO_4301) mimic the transcription pattern of the genes encoding thioredoxin-like enzymes instead of being induced at high salt. Moreover, the genes involved in glutathione synthesis were also unaffected by salt.

A clear pattern can be observed by glyoxalase (HELO_3670) and three glyoxalase-like proteins (HELO_3433, HELO_3142, HELO_2032) that show a consistent pattern of downregulation at high salt in comparison with both low and optimal salt.

Even though the genes for two peptide methionine sulfoxide reductases, *msrA2* and *msrB2*, were upregulated at low salt, those coding for another set of isoenzymes *msrA1, msrB1* as well as *msrC* did not show differential expression at different salt concentrations. MsrA and MsrB have been linked to the signal peptide system (SRP), guaranteeing a correct functioning of the SRP system to transfer proteins to the membrane (Weissbach et al., 2002). MsrA has also been linked to biofilm formation (Beloin et al., 2004).

#### 3.5.4. Iron Homeostasis

*H. elongata* has 27 genes with salt regulated homologues in *C. salexigens* involved in iron homeostasis. Four of these show differential expression at different salt concentrations: HELO_3026, for which the annotation in *H. elongata* has been updated to protease DegP1, is rather linked to protein folding stress. Gene *irgA* (HELO_3349) encodes a TonB dependent receptor, HELO_3347, which is part of an ABC transporter for iron, and *futA* (HELO_4001) is part of an iron (III) ABC transport system that is also downregulated at high salt while keeping similar levels of transcription under other conditions. Only one of the two paralogs to the σ-factor FecI in *C. salexigens*, csal_1098 had a homolog in *H. elongata* (HELO_2164) which was unregulated. The ferric uptake regulator HELO_4162 was not regulated either.

The ferric siderophore reductase in HELO_1154 follows exactly the same pattern as *futA* (HELO_4001). The *fetAB* operon (HELO_2138-9) also shows higher levels at low salt.

A cluster comprising the genes (HELO_3332-36), which encodes several enzymes related to siderophore synthesis and is co-located with a number of iron related genes, keeps similar levels of transcription at low and optimal salt, but is downregulated at high salt. A similar trend is followed by a neighboring cluster, a possible siderophore-iron ABC uptake system (HELO_3344-48) that includes three genes whose transcription correlates negatively with salt. The neighboring gene codes for a TonB dependent receptor (HELO_3339).

*H. elongata* has a number of TonB dependent receptors. Five of these (HELO_1153, HELO_2827, HELO_3304, HELO_3316 and HELO_3326) are transcribed in a similar pattern, namely more strongly at optimal salinity and weaker at low and high salinity. Additionally, the TonB dependent receptor HELO_3038 is upregulated at optimal salt with respect to both forms of salt stress while the *exbD* like gene HELO_3326 follows the opposite trend.

#### 3.5.5. Protein Folding Stress

Even though the sigma factor RpoH was upregulated at low salt, as has been observed in *C. salexigens* (Salvador et al., 2018), none of the heat shock proteins differentially expressed in such study was regulated by salt in *H. elongata*. The only gene encoding a heat shock protein that was found to be differentially expressed, *ibpA* (HELO_1663), had no homolog in *C. salexigens*. In this case, it can clearly be seen that the upregulation takes place only at low salt while the transcription levels for high and optimal salt are practically identical. All in all, of 15 homologues other than *rpoH* found in this group, only one was differentially expressed according to both methods applied: the protease gene *htpX* (HELO_2012), whose transcription shows negative correlation with salt.

Two additional proteases were found differentially expressed with confirmation from both methods: *degP1* (HELO_3026) that follows the general downward trend with salt and an S49 family peptidase with an opposite trend being actually downregulated at low salt. Two more genes encoding chaperones are also differentially expressed (HELO_2151D and HELO_1078) but their probable link to the usher pathway would explain their overexpression at low salt rather as a behavior consistent with the formation of pili and fimbriae.

#### 3.5.6. Chemotaxis and Motility

This functional group shows the clearest agreement between *H. elongata* and *C. salexigens*, being also one of the most clearly regulated modules among all studied. Of 41 homologous genes between both organisms, 38 are differentially expressed according to both methods and the other three are marked as differentially expressed by DESeq.

Chemotaxis genes are clearly downregulated with salt with some genes showing a correlation between salt and transcription and others keeping consistent levels at optimal and high salt while experiencing a sharp downregulation under low salt conditions. All the genes related to flagellar assembly and function have higher transcription levels at high salt with the detailed profile varying between a direct correlation with salt, upregulation at high salt or downregulation at low salt. In this regard, it is safe to assume that the behavior of flagella in *H. elongata* agrees with that studied in detail in *C. salexigens*.

In addition to the behavior already described for *C. salexigens*, several genes related to pili and fimbriae formation are differentially expressed. Three genes of the general secretion pathway *gspG, gspL*, and *gspE* (HELO_2777, HELO_2773, and HELO_2779, respectively) show higher transcription levels at high salt.

The above mentioned genes are the exception regarding transcription levels of pili and fimbriae related genes. Even though the genes in this category show a wide variety of patterns, most are expressed at higher levels at lower salt concentrations. This includes the gene *pilB* (HELO_2969) marked as upregulated at low salt by DESeq2, *pilP* and *pilN* also marked as upregulated at low salt by TPM count. Loci HELO_2151A-C, also show salt dependent TPM counts with higher transcription at low salt. In the case of HELO_2151A, the differential expression is also confirmed by DESeq. Although these genes are not clearly annotated, the COG database includes them in the fimbriae related COG5430 and they are part of a cluster that contains one more member of this COG as well as two genes related to the chaperone-usher pathway, which seems a good indication of the involvement of this cluster in fimbria formation.

### 3.6. Aerobic and Microaerobic Metabolism

The CRP family transcriptional regulator ANR (HELO_1634) showed a sharp downregulation at high salt. This regulator is homologous to *E. coli*'s FNR, which has been described as redox-sensing and activated by reduced levels of ATP to recover energy metabolism (Unden et al., 2002; Constantinidou et al., 2006; Batista et al., 2018). FNR is involved in controlling the metabolism and maintenance of the redox state by inducing pathways of anaerobic metabolism (Galimand et al., 1991; Winteler and Haas, 1996; Nikel et al., 2008; Tribelli et al., 2013). Because of this, FNR is strictly inactivated in aerobic conditions by directly reacting with oxygen molecules and subsequently losing its active dimeric state. The involvement of the FNR transcription factor in the regulation of respiratory genes during anaerobic growth and upon entering the stationary phase has also been reported for different *Pseudomonas* species (Ugidos et al., 2008; Tribelli et al., 2013, 2019). As has been noted before, the *nar* genes coding for nitrate reductase show a consistent trend toward higher transcription levels at low salt, just as the normally microaerobic *cydAB* genes. Finally, HELO_2706, which shows homology with the redox sensing two-component system RegAB, also shows decreasing levels as salt increased. HELO_2815 encodes a sensor histidine kinase, which is involved in motility suppression and biofilm formation, and shares this trend as well.

### 3.7. Ion Specificity of the ATPases

Two-sector ATPases (ATP synthases E.C. 7.1.2.2) are reversible *in vitro*. Bacterial / mitochondrial ATPases classified as F-type operate as ATP synthases *in vivo* while mostly eukaryotic V-type ATPases often operate as ATP driven ion transporters in physiological conditions. Even enzymes which function as ATP synthases are commonly referred as ATPases, and we will adopt this habit here. *H. elongata* has the gene clusters coding for two potential ATPases, one of the F-type and one of the V-type. One of the first prokaryotes discovered to possess a functionally expressed F-type and V-type ATPase simultaneously was *Enterococcus hirae*. In this bacterium, a proton gradient dependent F-type ATP synthase provides ATP while the Na^+^-translocating V-type ATPase is used for sodium homeostasis as a Na^+^ pump (Takase et al., 1993; Murata et al., 2001). V-type ATPases can also act as ATP synthases, like that of *Thermus thermophilus*, which is driven by a proton gradient (Nakano et al., 2008). In general, when two different ATPases occur in an organism, they have different ion specificities and operate in opposite directions (Becher and Müller, 1994), but cases have been reported where both enzymes act as ATP synthases (Becher and Müller, 1994).

To get a clearer picture about the ion specificity of the *H. elongata* F-type ATP synthase which has been annotated as H^+^-translocating so far, its c subunit was aligned with other F-type ATP synthases known for translocating sodium ions (Ferguson et al., 2006). The Na^+^-binding residues indicated with red boxes consisting of Q, E, and S do not exist in the *H. elongata* c subunit, confirming its current annotation (see Figure 9).

**Figure 8:**
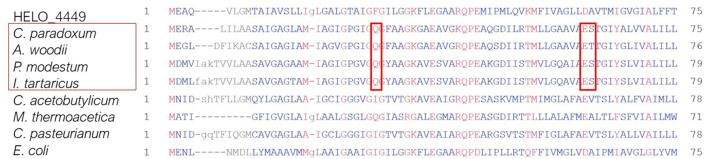
Alignment of various F-type ATP synthase c subunits for determination of the ion specificity for *H. elongata*. Commonly a glutamine (Q), glutamate (E) and serine (S) residue is needed for the binding of sodium which neither is present in the *H. elongata* F-type ATPase. The highest similarity was found with *E. coli* which is a proton translocating F-type ATP synthase.

The best studied Na^+^-translocating V-type ATPase is that found in *Enterococcus hirae* (EHR_08240). Murata et al. (2005) deciphered the relevant residues for ion binding in *E. hirae* (see Figure 8). For the binding of Na^+^ the residues E^139^, L^61^, T^64^, Q^65^, and Q^110^ are important. The E^139^ residue which is said to be essential for ion binding is conserved in the Na^+^-coupled version of *E. hirae* and also in *H. elongata*. But the remaining residues at positions 61, 64, 65, and 110 differ between *E. hirae* and the H^+^-coupled proteins. Interestingly, *H. elongata* shows traits of both versions as summarized in Table 3. At residues 64, 65, and 110, the ATPase in *H. elongata* has the same residues as that in *E. hirae*, but at residue 61 it is the same as in the H^+^-coupled ATPases. Murata et al. (2005) also reported that for Na^+^-binding the residues E^139^, T^64^, Y^68^, and Q^110^ are involved. The residue Y^68^ in the V-type ATPase of *E. hirae* is changed in *H. elongata* and a L is found instead even though the Y residue is conserved also for the H^+^ -coupled V-type ATPases.

**Table 3:** Overview of the essential residues for H^+^- and Na^+^-binding of the translocating subunit in V-type ATPases (Murata et al., 2005).

**Residue**	***E. hirae***	**H^**+**^-coupled**	***H. elongata***	***P. furiosus***	***A. xylanus***
139	E	E	E	E	E
61	**L**	**M**	**M**	L	L
64	T	I	T	T	T
65	Q	I	Q	Q	Q
110	Q	I	Q	Q	Q
68	**Y**	Y	**L**	Y	Y

*Notable differences from E. hirae (Na^+^) in H. elongata are marked ind boldface*.

**Figure 9:**
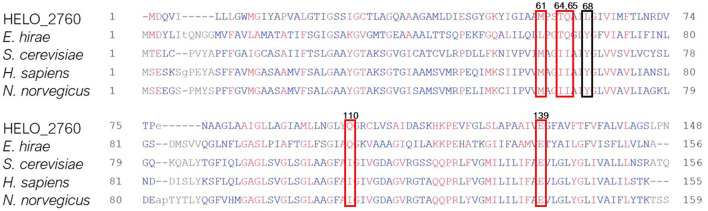
Alignment of various V-type ATPase K subunits/translocating units for determination of the ion specificity in *H. elongata*. Used are the same sequences as in Murata et al. (2005) which elucidated the Na^+^-translocating activity in *Enterococcus hirae*. In contrast, the remaining sequences refer to H^+^-linked V-type ATPases. Marked are the essential residues for H^+^- and Na^+^-binding (Murata et al., 2005). *H. elongata* shows similarities with both versions.

Furthermore, the analysis was extended to other V-type ATPases: the ATPase (Na^+^) of the hyperthermophile archaeon *Pyrococcus furiosus* (Mayer et al., 2012), the ATPase (Na^+^) of the halophilic alkaliphilic Gram-positive bacterium *Amphibacillus xylanus* (Kaieda et al., 1998), and the ATPase (H^+^) of *T. thermophilus* (Ferguson et al., 2006). *P. furiosus* and *A. xylanus* possess the exact same amino acid residues as *E. hirae* at the important residues for binding, in contrast to *H. elongata*. The *T. thermophilus* subunit did not match with any of the previously mentioned sequences. With only 99 aa the protein is much shorter and has no match for E^139^ residue stated to be essential for ion binding could be found. All in all, the subunit was much more similar to the H^+^-translocating c subunits of F-type ATP synthases investigated above (see Figure 9).

### 3.8. Acidity of the Proteome

It is well established (Reistad, 1970) that halophilic bacteria and archaea that accumulate high concentrations of KCl in their cytoplasm, which is often called the salt-in strategy, have an over-abundance of acidic amino acids in their proteins. Further analyses have shown that moderately halophilic bacteria have levels of acidity in their proteomes that are in-between those of halophiles and non-halophiles (Oren, 2013). One such case is *H. elongata* (Gandbhir et al., 1995), which prompted the hypothesis that these bacteria also accumulate salts in the cytoplasm to a certain extent. Analysis of 238 proteins from the genome of *C. salexigens* questioned this by indicating that the acidity of the proteins within this sample may be limited to periplasmic proteins (Oren et al., 2005).

By assigning subcellular locations to proteins using the psort database (Peabody et al., 2016), we could calculate the distribution of acidic and basic amino acids in the cytoplasmic proteins of a number of bacteria and archaea. The organisms were chosen due to their different strategies. *E. coli* (Riley et al., 2006) and *Pseudomonas putida* (Belda et al., 2016) are non-halophilic bacteria. *Alteromonas macleodi* (Ivars-Martinez et al., 2008) is a marine bacterium. *C. salexigens* (Copeland et al., 2011) is a close relative of *H. elongata* and both are considered moderately halophilic. *Halobacterium salinarum* (Pfeiffer et al., 2008) is an extremely halophilic archaeon and *Salinibacter ruber* (Pena et al., 2010) a bacterium that shares its same habitats, both organisms follow the salt-in strategy. Finally, *Halorhodospira halophila* (Challacombe et al., 2013) is an extremely interesting example because it can accumulate high concentrations of KCl in its cytoplasm (up to 35 %) but it can also survive with a much lower amount of just 5 % when it grows at low salinity. This shows that the salt-in strategy does not only lead to obligate halophiles (Deole et al., 2013).

**Figure 10:**
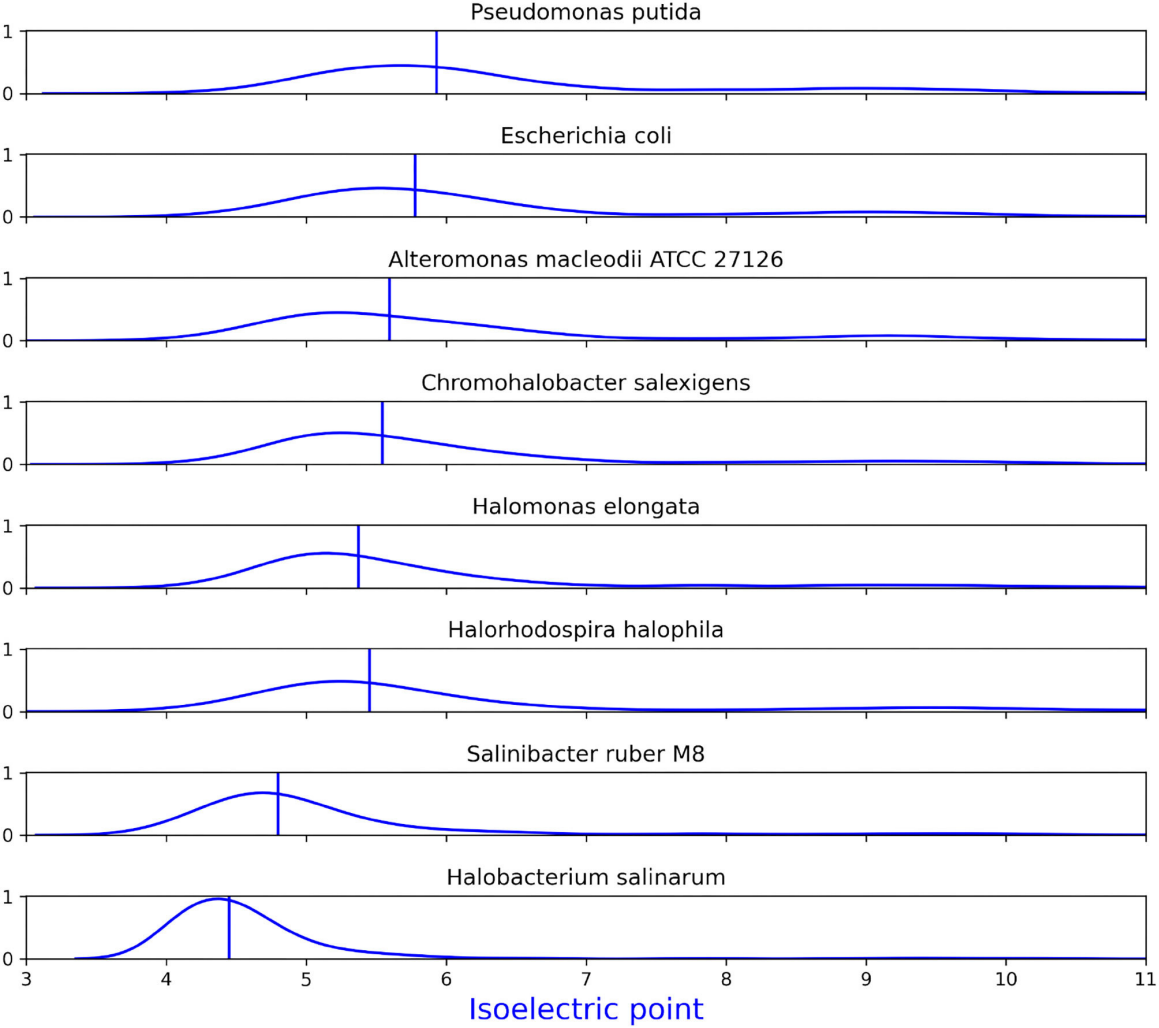
Distribution of isoelectric points in the cytosolic proteins of several prokaryotes as density plots. Vertical lines show the median for each plot. The amount of genes to which psort could assign a reliable location varies from one bacteria to another as does the fraction of this that were classified as cytosolic. The amount of proteins identified as cytosolic was: *Pseudomonas putida*: 2457, *Escherichia coli*: 1935, *Alteromonas macleodii* ATCC 27126: 1616, *Chromohalobacter salexigens*: 1726, *Halomonas elongata*: 1958, *Halorhodospira halophila*: 1301, *Salinibacter ruber* M8: 1358, *Halobacterium salinarum: 1250*.

Figure 10 shows the distribution of isoelectric points of the cytosolic proteins of these microorganisms. The medians show a trend toward higher acidity as we move from non-halophiles to marine bacteria, moderate halophiles and extreme halophiles. It is also noteworthy that the distributions of acidic and basic amino acids follow different trends. While the fraction of acidic amino acids per protein follow a very similar trend to that of the isoelectric points. The fraction of basic amino acids shows reduced variability and even a different direction between the displacement of the median toward more basic amino acids in moderate halophiles and toward less basic amino acids in extreme halophiles (see Supplementary Figure S2).

### 3.9. Optimal Salinity vs. Salt Related Stress

Since both the high and low-salt environment reduce the growth rate with respect to the optimal salt concentration, both conditions can be assumed to induce stress in some form. A comparison was made between the transcription levels in TPM counts in order to find out which genes are up or downregulated on both cases with respect to the optimal conditions. A total of 38 genes are upregulated by salt related stress caused by either high or low salt concentration. A total of 104 genes were downregulated.

Among the 38 genes that were upregulated by salt related stress, there were 10 ribosomal or ribosome associated genes. Among the others we can find one gene of the Mrp potassium-proton exchanger (HELO_3518), enzymes HELO_1780 and HELO_1781 that convert D-galactonate to Glyceraldehyde-3-Phosphate, a GntR family transcription regulator HELO_1775, TRAP transporter large transmembrane protein HELO_1778 (belongs to COG4664 mannitol/chloroaromatic compounds transport), ExbD family protein HELO_3038, heme exporter protein CcmD HELO_3727 and the cytochrome bo′ CyoD.

A much larger number of genes was downregulated with either stress condition. First, we will discuss those genes which share a pattern of differential expression between optimal and not optimal salt concentration and also show different levels of expresssion on both extremes. The two genes HELO_2483-4 are part of an ABC transporter. These genes show their highest transcription levels at optimal salt but are still more expressed at high than at low salt (LFC2 ≥ 1.5). The probable substrate suggested by COG is glycerol-3-phosphate. There are also nine genes that present a transcription pattern that is a mirror image of this, with higher levels at low salt. Among these are the alkaline phosphatase HELO_1560, ABC-type transporter genes HELO_3005-6 with a probable substrate of phosphate/phosphonate, an SSS family transport protein, probable substrate acetate and TRAP transporter substrate-binding protein with probable substrate mannitol/chloroaromatic compound. All these probable substrates are predicted based only on the COG into which they are classified.

Among the genes that show very similar levels of transcription on both sides of the salt spectrum, the COG class G (carbohydrate transport and metabolism) is clearly dominant with 39 genes. This includes glucokinase (HELO_3629), phosphoglucoisomerase (HELO_4245), pyruvate kinase (HELO_4243), transketolases (HELO_1184 and HELO_1196), transaldolase HELO_3967, alpha-glucosidases (HELO_3677, HELO_3687) and the sucrose (HELO_3673) and maltose (HELO_3682) porins. The remainder of the genes in this COG class are diverse transporters. Also following this pattern of regulation but not linked to carbohydrate metabolism and transport there are amino acid transporters such as: LysE family transport protein HELO_2956, ABC amino acid transporter AapPQM (HELO_4316-9), and ABC transport permease protein HELO_1115. and genes belonging to other classes.

It is well known that bacteria growing on different carbon sources, tend to have a ribosomal fraction of the proteome that correlates with growth rate (Scott et al., 2010). Here, we observe the opposite trend where a decrease in the growth rate due to salt, results in a shift of the proteome from carbohydrate metabolism to ribosome production.

### 3.10. Ectoine Excreting Strain

As mentioned above, strain KB2.13 (Δ*teaABC*Δ*doeA*) lacks the ectoine uptake system that recovers ectoine from the periplasmic space through symport with sodium. This results is constant loss of ectoine to the medium that defines the ectoine excreting phenotype. The transcriptional profile in this mutant differs from the wild type in a similar way as low salt transcriptome compares to high salt, albeit with a much smaller number of regulated genes. The removal of the *teaABC* cluster results in overexpression of the fourth gene and regulator *teaD*, because it is now next to two strong promoters. The fact that this gene is down-regulated at low salt serves as a control to differentiate the effects of altering the levels of *teaD* regulator from those of the removal of the ectoine Na^+^ uptake.

**Figure 11:**
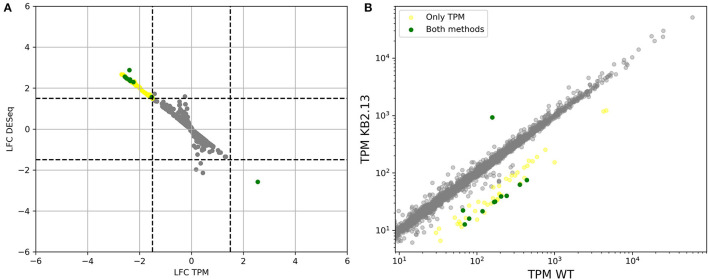
**(A)** Comparison of log2-fold-changes between wild type and strain KB2.13 obtained using TPM count vs. DESeq. Dashed lines mark the threshold for differential expression. **(B)** Comparison of TPM counts between wild type and strain KB2.13. Genes that are considered to be differentially expressed by both methods are plotted in green. Only by DESeq in orange and only by TPM count in yellow.

The number of regulated genes between the wild type and strain KB2.13 is much smaller than between any other sample but also shows clear results (see Figure 11). Only one gene is shown to be upregulated in strain KB2.13, the above mentioned *teaD*, whose transcription is now under direct control of the promoter of the *teaABC* operon. On the other hand, fifteen genes are clearly down-regulated in the mutant according to both DESeq and TPM count. These genes are the four genes deleted in the mutant, the second gene in the ectoine degradation pathway, *doeB*, and ten flagellar structural genes.

**Figure 12:**
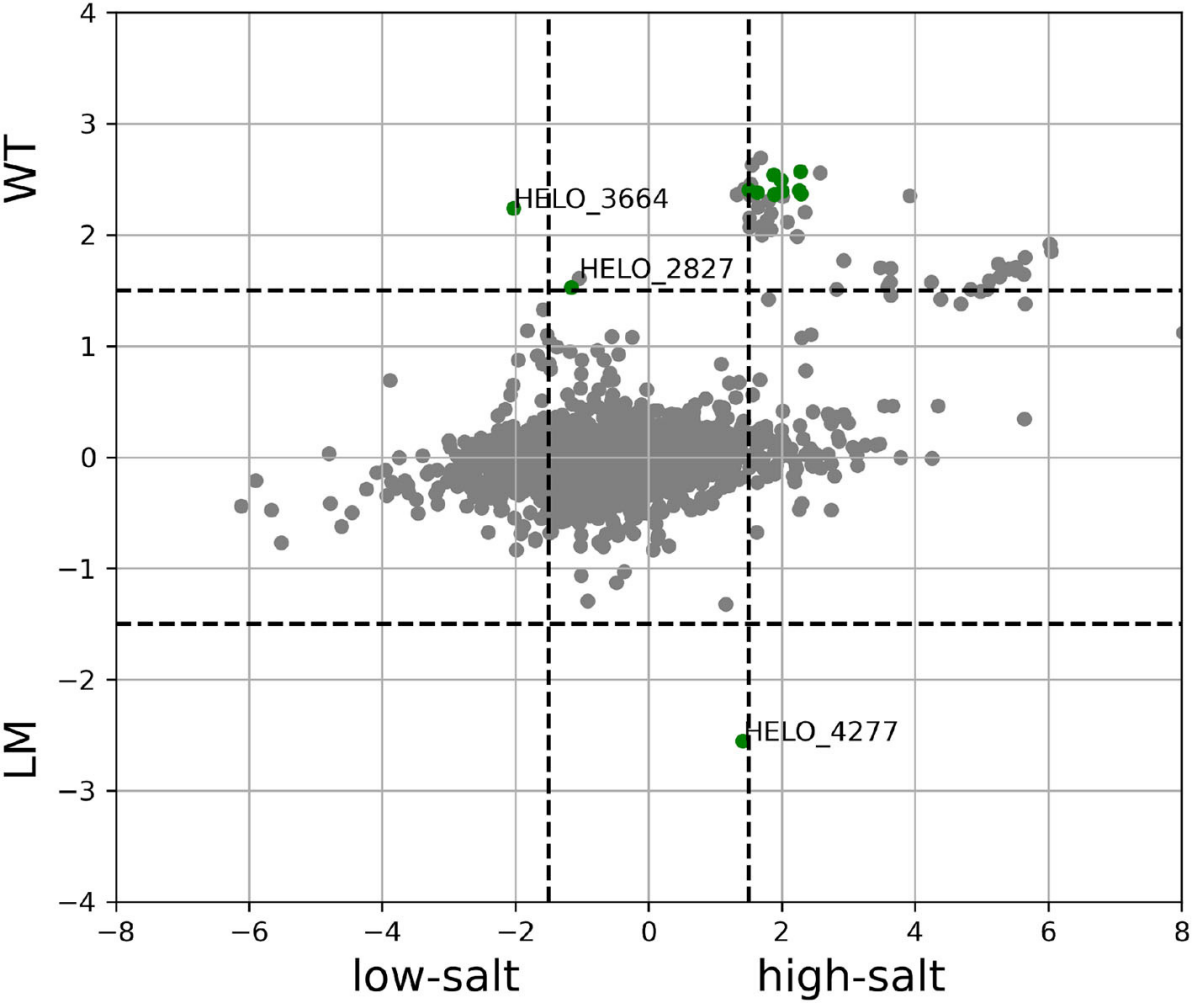
Difference in transcription levels between low and high salt (*x*-axis) and between strain KB2.13 and wild type (*y*-axis) both in log2-fold-change. Genes in green are those marked as differentially expressed according to both DESeq and TPM count.

While DESeq did not identify any other differentially expressed genes, there was a total of 41 additional genes that were above the threshold chosen for differential expression in TPM counts. Three of these genes are uncharacterized proteins (HELO_4350, HELO_4351, and HELO_4347), 23 are flagellar genes, 10 were involved in chemotaxis and finally, HELO_2827 encodes a TonB dependent receptor. The correlation between the response to the mutations and to a low-salt environment is clear as can be seen in Figure 12.

## 4. Conclusions and Discussion

Even though it has attracted more attention than any other adaptation mechanism, recent studies point at ectoine accumulation as only a part of a far wider systemic response. After the sequencing (Schwibbert et al., 2011) and a careful reannotation (Pfeiffer et al., 2017) of its genome, the metabolic capabilities of *H. elongata* are well known. Among these capabilities there is a great variety of alternative modes of operation based on the presence of different pathways that can fulfill similar roles. In this work, we aimed at obtaining further insight on how these capabilities are actually put to use by this bacterium and get a dynamic picture on how it adapts to different environments, specially to varying salinity.

It has been previously suggested that the glycolytic flux in *H. elongata* is mainly channeled through the Entner-Doudoroff pathway, with the Entner-Meyerhoff-Parnas pathway being mostly involved in gluconeogenesis thanks to the reversibility of the PPi-dependent phosphofructokinase (PFK) in *H. elongata* (Kindzierski et al., 2017). Even though there is no regulation of the PFK at the transcriptomic level, the genes involved in cytoplasmic glucose utilization branching toward the ED pathway *via* glucose-6-phosphate dehydrogenase and the genes encoding the ED pathway themselves were strongly downregulated during gluconeogenetic growth. This supports the notion that *H. elongata* relies on the ED pathway for glucose metabolization. Furthermore, up-regulation of a glyceraldehyde 3-phosphate dehydrogenase under growth on acetate also supports a possible gluconeogenic role of the EMP pathway.

The availability of transcriptional data from *C. salexigens*, a close relative of *H. elongata* from a similar ecological niche, provides an excellent context to analyze our results (Salvador et al., 2018). Both microorganisms seem to have an identical response in terms of flagellar assembly and chemotaxis. Furthermore, the way this same response is present in the mutant lacking the ectoine-sodium symporter TeaABC raises interesting questions on how this response is triggered. Other metabolic responses alternate general similarities with important differences, as can be seen in the metabolism of compatible solutes. Both organisms react similarly in terms of ectoine and, to a certain extent, betaine metabolism, but unlike *C. salexigens, H. elongata* does not show regulation by salt in the transcription of *lysC* (HELO_3742), which encodes the only aspartokinase in its genome. This confirms previous observations (Kindzierski et al., 2017) and complements the observed differences reported at the biochemical level (Hof, 2011). Aspartokinase is normally an important control point for the synthesis of amino acids of the aspartate family and it was found to be regulated by salt in *C. salexigens* but these pathways seem to follow a completely different control pattern in *H. elongata*. Oxidative phosphorylation shows a common response to the extent in which the normally microaerobic bd quinol oxidase is activated but no regulation was observed in *H. elongata* for complexes that reduce ubiquinone.

Another significant difference is that *H. elongata* does not seem to be experiencing protein folding and oxidative stress. The only transcriptional response in this sense seems to be the upregulation of one of the two sets of methionine sulfoxide reductases: MsrAB2, which seem to play very specialized roles in other bacteria rather than being a general repair mechanism (Weissbach et al., 2002; Beloin et al., 2004).

Iron homeostasis seems to show mostly a downregulation of uptake mechanisms at high salt and maybe some TonB receptors going up at optimal salinity. These results are consistent with the observations in *C. salexigens*, where iron uptake was shown to be higher at low salt. This would be a logical consequence of the solubility of iron (III) in a NaCl solution. It has been shown that solubility has a minimum at an ionic strength of 0.6 M (Liu and Millero, 2002), so it would be expected that the solubility of iron decreases with salt for non-halophilic bacteria but it increases for halophilic bacteria growing at high salinity. Thus, it is not surprising that genes involved in siderophore synthesis and iron uptake mechanisms decrease their transcription levels at high salt in *H. elongata* and *C. salexigens* but the trend is reversed in *Bacillus subtilis* (Hoffmann et al., 2002). The transcriptional response of *C. salexigens* itself was not as clear as that in *H. elongata*, showing some iron transporters and siderophore synthesis related genes increase their transcription level at high salinity and others at and low salinity. A possible answer to such an apparently conflicting transcriptional response could be seen from the behavior of some of these genes at optimal salt in *H. elongata*. Some genes such as HELO_3038, paralogs to the *tolQR* operon show upregulation under both forms of salt stress while the TonB dependent receptor HELO_3326 showed up-regulation at optimal salt. A trend to increase or decrease expression with stress could appear as up(down)regulation by salt if only the extreme cases are compared if the level of stress is not exactly the same on both sides of the spectrum.

All in all, the behavior observed here, as well as that reported in previous studies both theoretical and experimental, is consistent with an inability of *H. elongata* to decrease its cytoplasmic sodium level low enough to create a significant sodium-motive force at 0.17 M NaCl. First of all, the absence of a strong sodium-motive force would explain previous experimental results where the sodium coupled anaplerotic reaction catalyzed by oxaloacetate decarboxylase (Oad) fails to sustain growth at 0.17 NaCl (Hobmeier et al., 2020). The failing Na^+^ gradient must be either necessary for direct ATP production by the Na^+^-translocating V-ATPase or to top up the proton-motive force through the sodium-proton exchangers. The depolarization of the membrane suggested by the expression of the UPF0057 family protein HELO_2679 would suggest the latter. Either the low membrane potential, the insufficient ATP production or both are good suspects for causing the overexpression of the normally microaerobic electron transport chains encoded in the *cyd* and *nar* clusters. The lack of these responses or indicators of low membrane potential in *C. salexigens* are also to be expected, since this microorganism does have a NDH-1 able to build a proton-motive force without any help from the sodium gradient. The case could be made for an increase of membrane permeability to Na^+^ as the cause for the drop in sodium-motive force. While possible, this mechanism would very likely affect the proton gradient as well and create difficulties for both microorganisms, eliciting a more similar response in both. Furthermore, the up-regulation of the Na^+^ pumping ATPase would be expected to counter the leak. The overall transcription pattern is rather compatible with a problem originating in the gradient itself, rather than in the flux needed to create or maintain it. This scenario also explains PHB production as a way to store the carbon that cannot be used for growth due to a low energy production and reduction equivalents that cannot be efficiently used by the electron transport chain. It is noteworthy, that sodium concentrations in the closely related organism *C salexigens* remain constant at different medium salinities (Salvador et al., 2018), which is consistent with our hypothesis for *H elongata*. Sodium measurements are not done as frequently in halophiles as would be desirable due to the methodological difficulties to avoid contamination from the medium or to differentiate between bound and free sodium, cytoplasmic vs. periplasmic, etc. The accumulation of indirect evidence such as mentioned here, however, should be taken as an incentive to tackle these issues in the future.

This idea of high cytoplasmic sodium levels is further backed by the acidity of the proteome. Specially since the fraction of acidic amino acids in *H. elongata* is as high as that of a (non-obligate) salt-in organism such as *H. halophila*. Adding the observed accumulation of potassium (Kraegeloh and Kunte, 2002) and a proposed high concentration of sodium in the cytoplasm would also explain how *H. elongata* withstands much higher salinities than non-halophilic bacteria supplied with ectoine. Would this be confirmed, it would mean that salt tolerance by *H. elongata* follows a mixed strategy were potassium and, to a lesser extent, sodium accumulation provides a basal resistance to (and dependence from) salt that is then topped up by ectoine when salinity increases beyond a threshold.

## Data Availability Statement

The RNAseq data generated in this study has been uploaded to SRA and can be accessed under the accession number PRJNA803715.

## Author Contributions

AM-S, AK, and HK: obtained the funding. HK, FP, KH, and AM-S: conceptualized the research. AM-S, KP-G, and KH: supervised the work. KH and QN: carried out the experiments. MC, KH, and AM-S: wrote the software and analyzed the data. AM-S: wrote the first draft of the manuscript. KH, FP, and HK: participated in the elaboration of subsequent versions. All authors read and approved the final version of the manuscript.

## Funding

This work was funded by the German Federal Ministry of Education and Research (BMBF) through project HOBBIT (031B03).

## Conflict of Interest

The authors declare that the research was conducted in the absence of any commercial or financial relationships that could be construed as a potential conflict of interest.

## Publisher's Note

All claims expressed in this article are solely those of the authors and do not necessarily represent those of their affiliated organizations, or those of the publisher, the editors and the reviewers. Any product that may be evaluated in this article, or claim that may be made by its manufacturer, is not guaranteed or endorsed by the publisher.
