# Supplementary figures and images for: Adaptation to Varying Salinity in Halomonas elongata: Much More Than Ectoine Accumulation
Source: Front Microbiol. 2022 Mar 30;13:846677. doi: 10.3389/fmicb.2022.846677 (PMC9006882; doi:10.3389/fmicb.2022.846677)

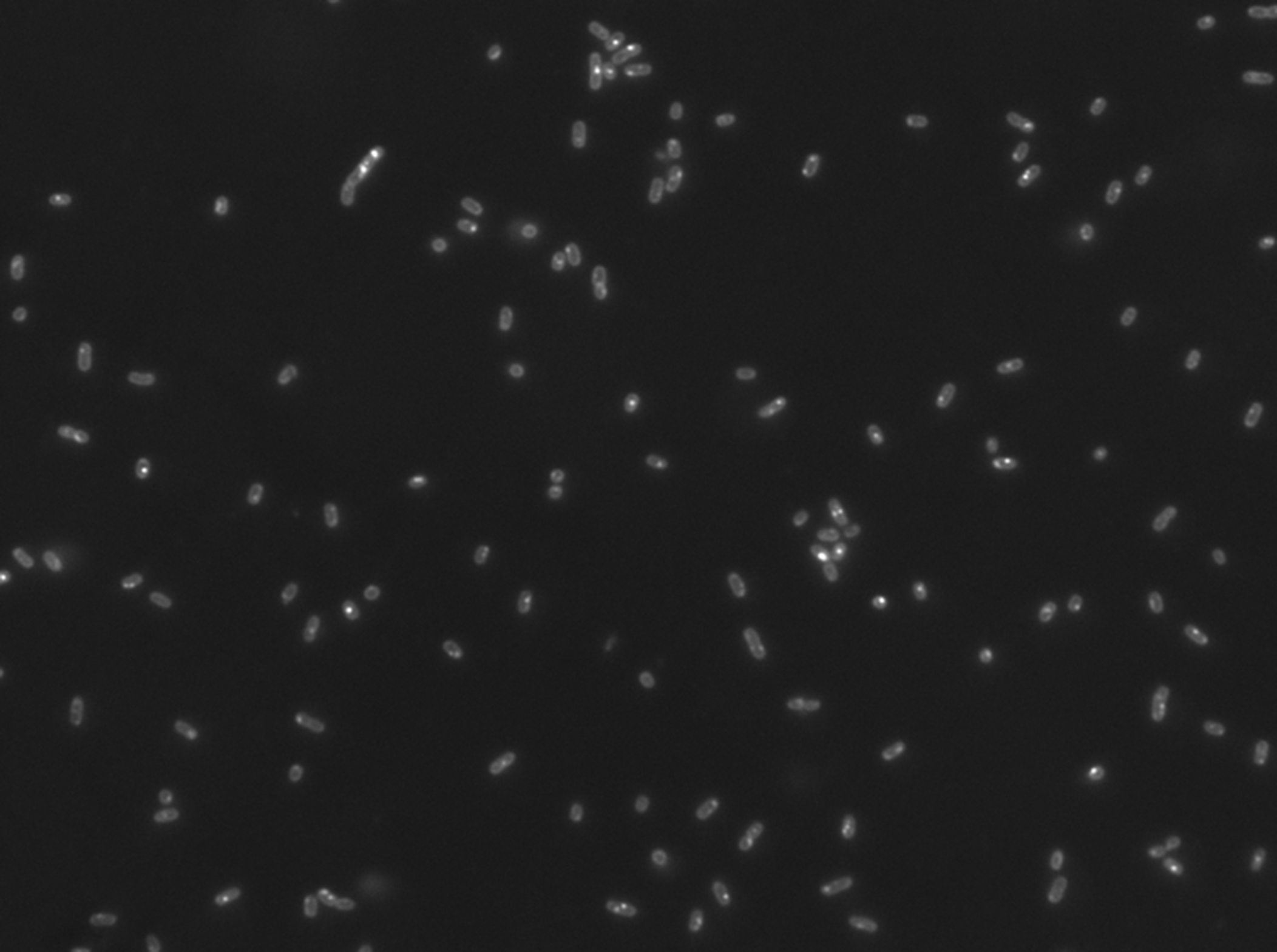

Supplement: Supplementary file 6 [file Image_1.JPEG]

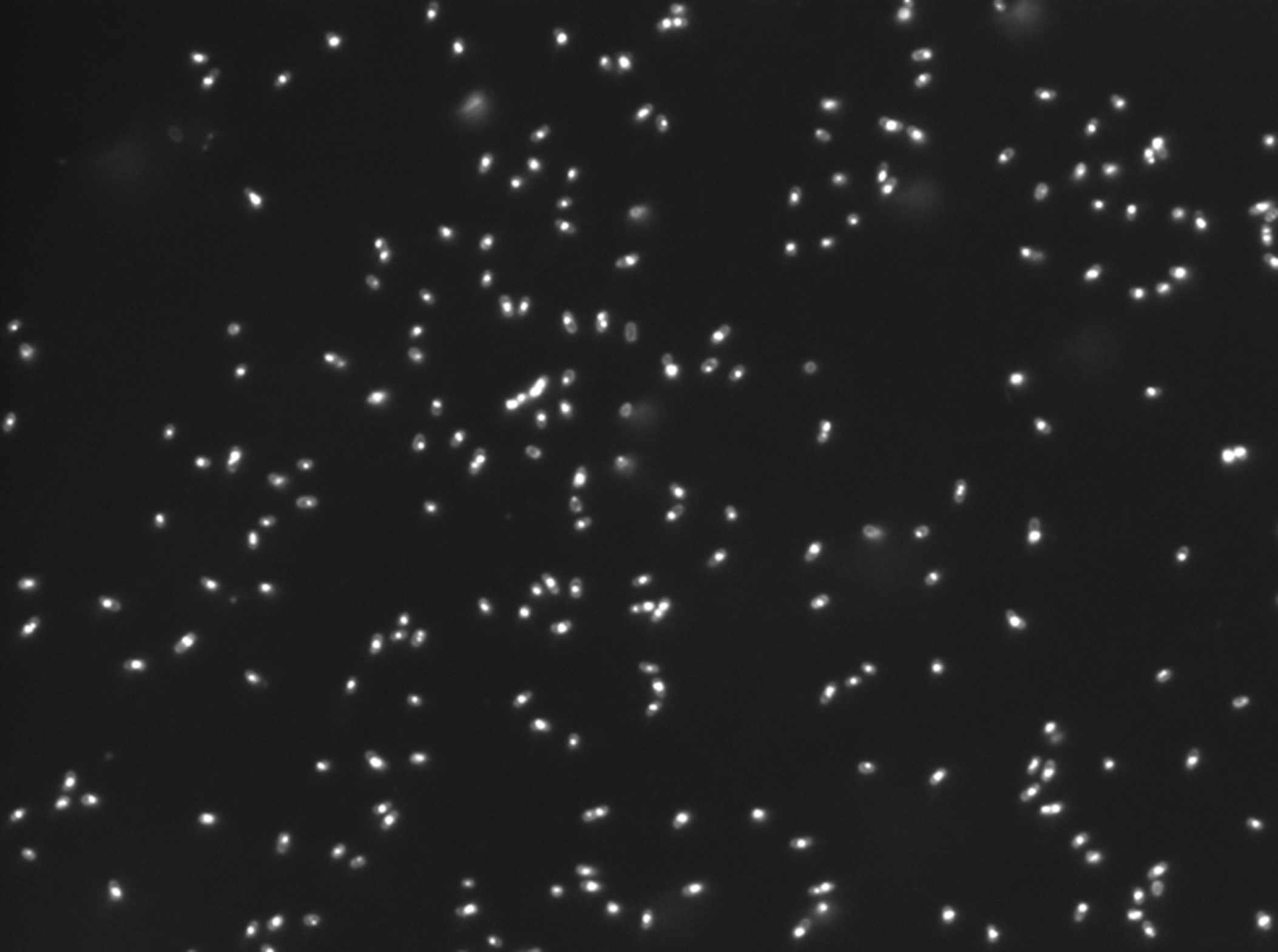

Supplement: Supplementary file 7 [file Image_2.JPEG]

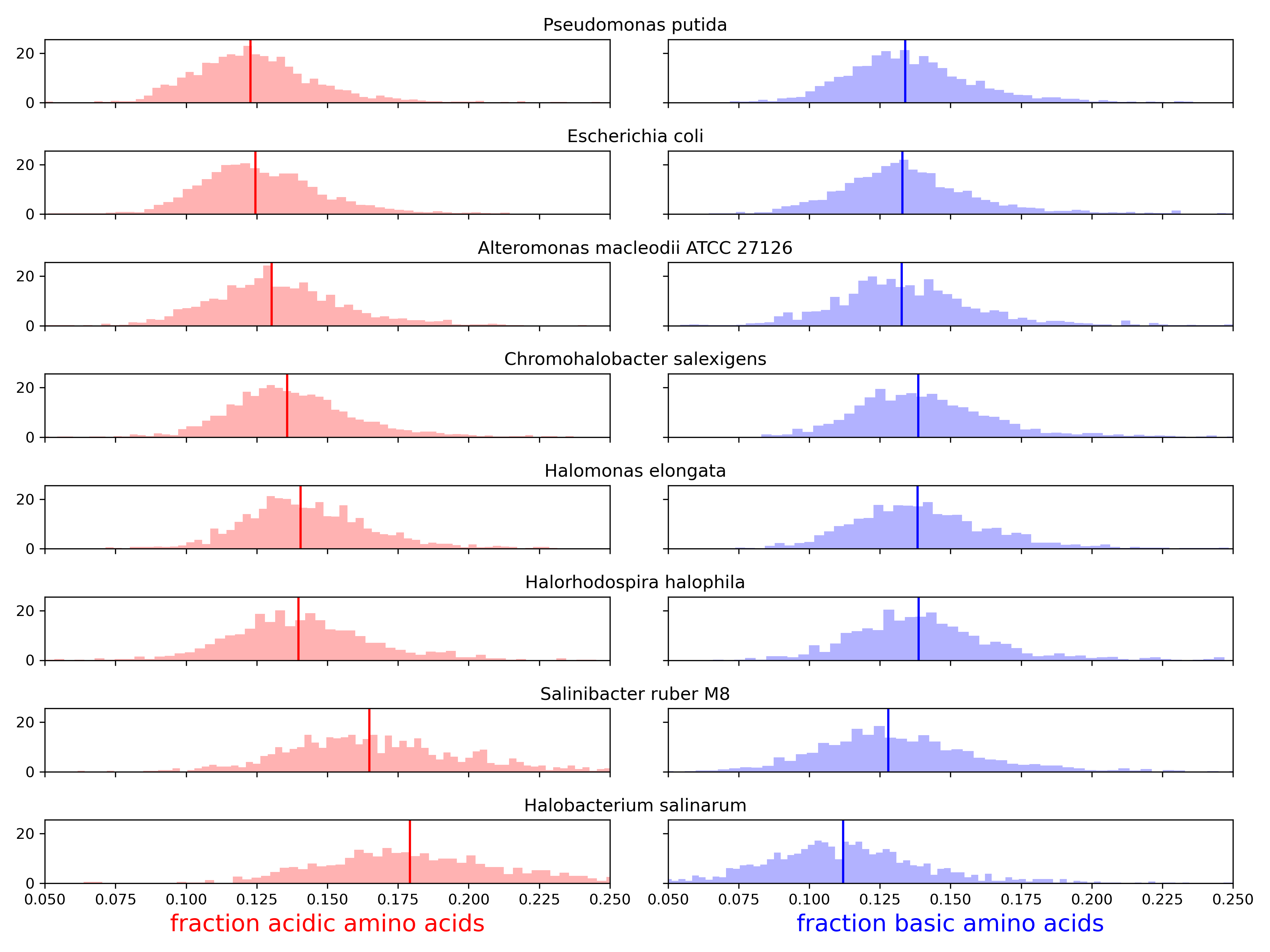

Supplement: Supplementary file 8 [file Image_3.PNG]
